# Dengue Myocarditis Complicated by COVID-19: A Case Report on Dual Viral Infection

**DOI:** 10.7759/cureus.17076

**Published:** 2021-08-10

**Authors:** Samiksha Gupta, Shreya Arora, Monica Gupta, Gautam Jesrani

**Affiliations:** 1 General Medicine, Government Medical College and Hospital, Chandigarh, IND

**Keywords:** acute undifferentiated febrile illness, co-infection, covid-19, dengue fever, myocarditis

## Abstract

During the ongoing pandemic of coronavirus disease 2019 (COVID-19), it is crucial for clinicians to have an insight into the emerging co-infections. As the dengue virus (DENV) and severe acute respiratory syndrome coronavirus 2 (SARS-CoV-2) have related symptomatology, a high index of suspicion is required for prompt diagnosis of concurrent infections involving these two pathogens, especially in the areas endemic for tropical diseases, i.e., dengue fever (DF), malaria, enteric fever, chikungunya, leptospirosis, etc. In this report, we present the case of a middle-aged man from Northern India, who had DF with myocarditis, and was simultaneously found to have COVID-19 co-infection. The patient was managed as per the COVID-19 protocol and had a favorable outcome.

## Introduction

Novel coronavirus disease 2019 (COVID-19), caused by severe acute respiratory syndrome coronavirus 2 (SARS-CoV-2), has resulted in a lethal global pandemic [1]. It exhibits varied clinical manifestations, potentially affecting every organ system, although fever remains a common symptom [2]. In India, dengue virus (DENV) infection, transmitted by *Aedes aegypti*, is one of the most common causes of acute undifferentiated febrile illness (AUFI) with endemicity, usually during the monsoon and post-monsoon seasons. The symptom profile consists of mild non-specific symptoms such as fever, malaise, and vomiting, making the clinical recognition of the disease laborious [3]. In rare cases, myocarditis can also be seen in the disease course of dengue fever (DF). Concurrent infection of COVID-19 and dengue myocarditis is rarely described in the current literature, and in this report, we describe one such presentation. Additionally, we review and describe the overlapping symptomatology in such co-infections, which is imperative for clinicians to recognize in clinical practice.

## Case presentation

**Figure 1:**
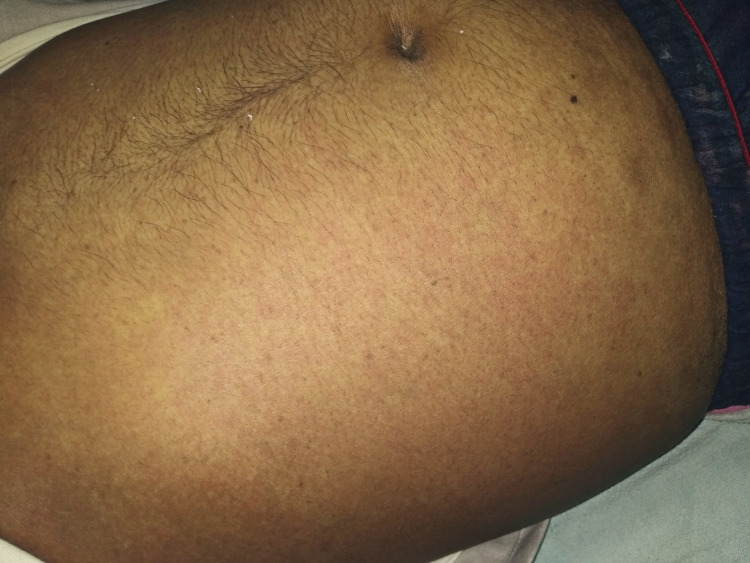
Skin rashes on the abdomen of the patient on presentation

**Figure 2:**
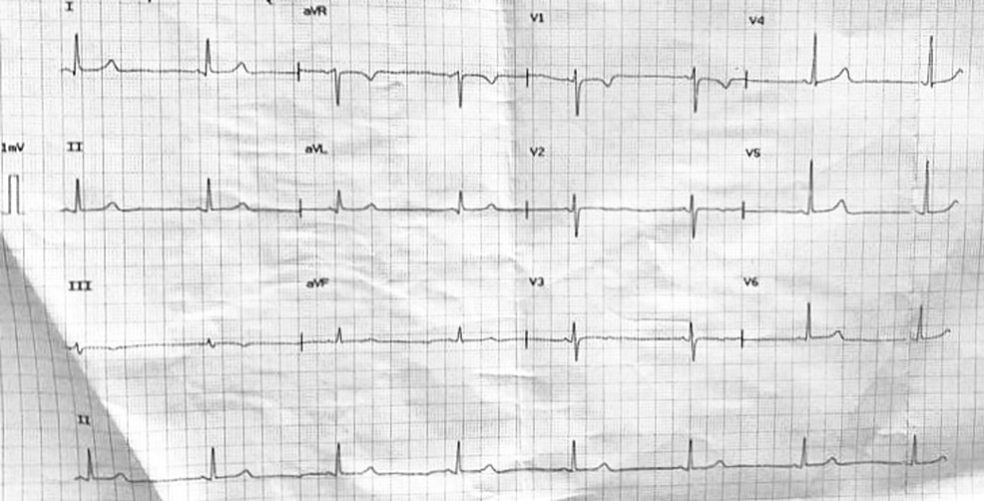
The electrocardiogram of the patient demonstrating sinus bradycardia on presentation

A 65-year-old man with no known comorbidities in the past presented to the emergency department with a history of high-grade fever and chills for four days, which was relieved with antipyretic medication. He also had a history of intermittent episodes of the passage of reddish urine, lasting for two days, which was not associated with abdominal pain or dysuria. At presentation, he had non-productive intermittent bouts of cough but denied any exposure to COVID-19 or tuberculosis patients. On examination, the patient had a temperature of 38.5 ºC, a regular pulse rate of 48 beats/minute, blood pressure of 90/64 mmHg, respiratory rate of 18/minute, and was maintaining an oxygen saturation of 97% on room air. He also had a generalized erythematous rash over the abdomen and lower limbs (Figure 1). The cardiovascular and respiratory examination did not reveal any abnormality. His electrocardiogram demonstrated sinus bradycardia (Figure 2), but the chest radiograph revealed no cardiomegaly or lung pathology on presentation.

On investigations, his complete blood profile revealed a total leucocyte count of 2.3 x 10^9^/L with 55% neutrophils and 40% lymphocytes and thrombocytopenia of 20 x 10^9^/L (Table 1). In light of the thrombocytopenia, the patient was evaluated for malaria, scrub typhus, leptospirosis, enteric fever, and DF. DENV serology for immunoglobulin M (IgM) came out to be positive with test levels of 0.72 optical density (OD, cut-off value: 0.31) while DENV NS-1 antigen was negative by enzyme-linked immunoassay. Thrombocytopenic measures were advised (i.e., to avoid brushing teeth or straining and no intramuscular injection) along with intravenous fluid therapy. Due to relative bradycardia, the possibility of viral myocarditis was considered. His qualitative troponins were negative; however, creatine phosphokinase-MB (CPK-MB) was considerably high, i.e., 72 U/L (cut off value: <25), and N-terminal pro-B type natriuretic peptide (NT-proBNP) was 1560 pg/mL (cut off for age: <900 pg/mL). 2D-echocardiography showed normal left ventricular systolic function, grade-I diastolic dysfunction, and preserved ejection fraction of 64%. Regarding hematuria evaluation, his ultrasound of the renal system did not reveal any anatomical discrepancy or stones within the pelvicalyceal system and therefore it was attributed to severe thrombocytopenia.

**Table 1:** Important blood investigations of the patient during the hospital course

Investigations	Normal range	Day 2	Day 13
Hemoglobin (g/dL)	12.0-16.0	13.0	13.4
Total leucocyte count (x 10^9^/L)	4.0-11.0	2.3	5.7
Neutrophil-lymphocyte ratio	1-3	1.4	1.8
Platelet count (x 10^9^/L)	150-450	20	178
Aspartate transaminase (AST) (U/L)	5-40	187	54
Alanine transaminase (ALT) (U/L)	5-35	46	40
Creatine phosphokinase-MB (U/L)	<25	72	31
C-reactive protein (CRP) (mg/L)	0-5	49	12
Procalcitonin (PCT) (ng/mL)	<0.05	0.13	0.03
D-dimer (µg/mL)	<0.5	0.8	0.4
Lactate dehydrogenase (LDH) (U/L)	235-470	569	480
Interleukin-6 (pg/mL)	4-12	12.2	5.0
Ferritin (ng/mL)	22-322	453	327

**Figure 3:**
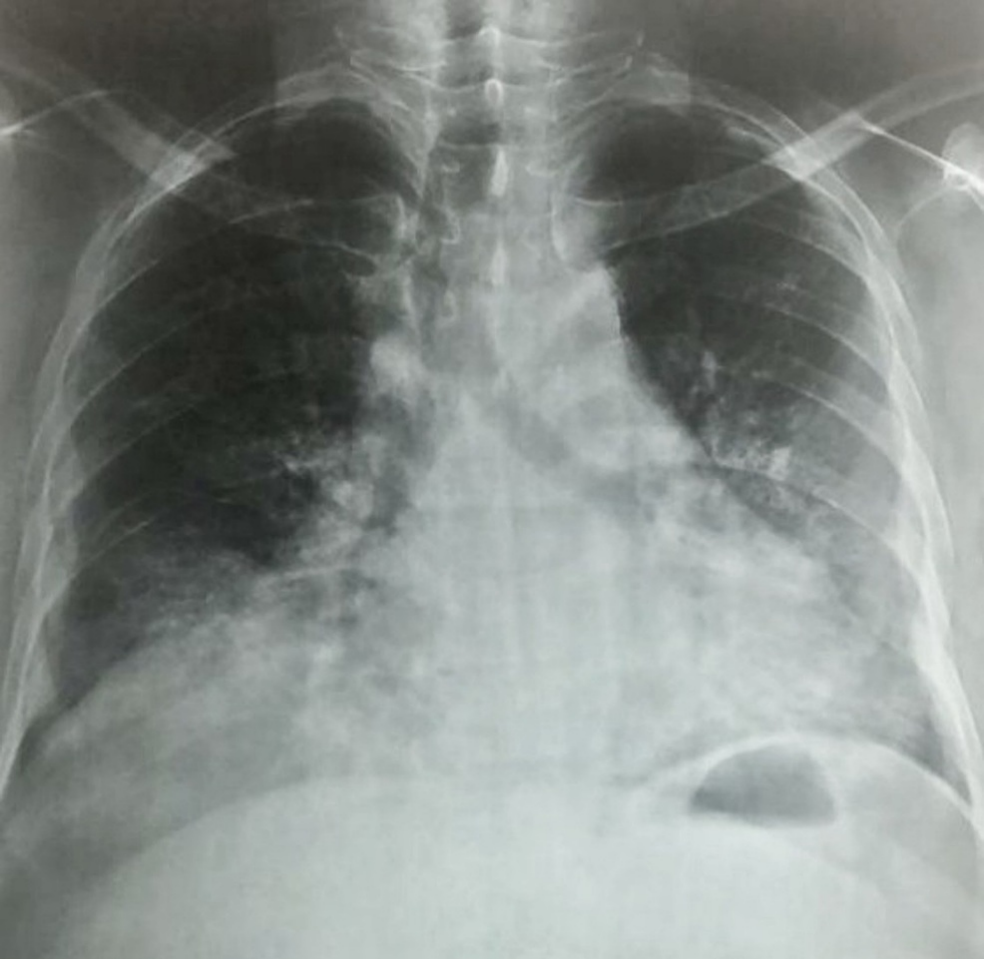
Subsequent chest X-ray of the patient demonstrating consolidation in the left middle zone and bilateral lower zone

**Figure 4:**
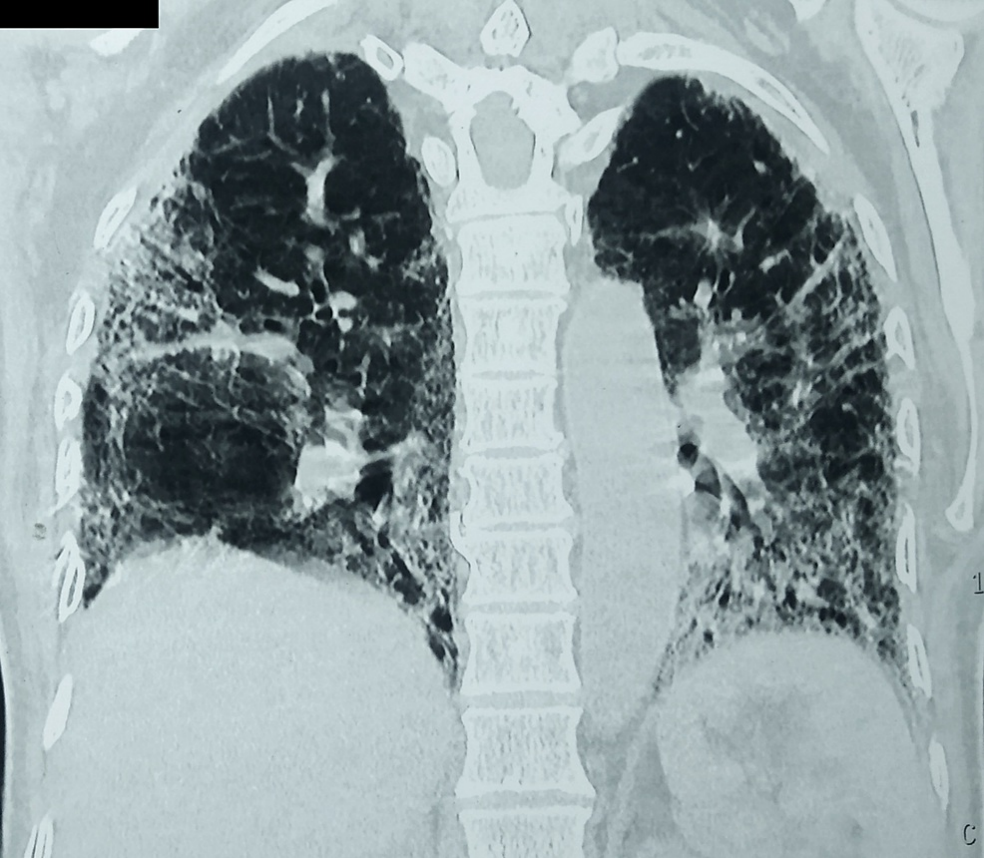
Coronal section of the high-resolution CT of the chest demonstrating bilateral ground-glass opacities CT: computed tomography

On the fifth day of hospitalization, he developed breathlessness with a respiratory rate of 26/minute and a saturation of 90% on room air following which a repeat chest radiograph was performed, which suggested bilateral heterogeneous opacity in lower zones (left > right) and the left middle zone (Figure 3). He was managed with oxygen supplementation through a venturi mask and had a normal oxygen saturation thereafter. High-resolution CT (HRCT) of the chest was done, which was suggestive of bilateral ground-glass opacities (left > right) and a severity index of 13/25 (Figure 4). In light of the ongoing pandemic and local protocol, the patient was tested via nasopharyngeal swab by reverse transcriptase-polymerase chain reaction (RT-PCR) for COVID-19, and he was confirmed positive for SARS-CoV-2 virus.

Only intravenous steroids (0.1 mg/kg dexamethasone for seven days) could be given since anticoagulation was omitted due to thrombocytopenia. From day three onwards, the patient had gradual improvement in the heart rate, platelet count, and continued to have stable blood pressure. The patient was discharged successfully after 17 days in the hospital isolation facility with no residual symptoms on follow-up.

## Discussion

Numerous concerns have been raised regarding the co-infection of COVID-19 with endemic illnesses such as DF, scrub typhus, enteric fever, and leptospirosis. One of them pertains to similar presenting complaints like fever, malaise, headache, abdominal pain, or vomiting. Uncertainty and delay in diagnosis usually ensue due to the confounding clinical picture and, additionally, related to laboratory investigations for leucopenia and thrombocytopenia. Such co-infections add an additional burden to the already over-worked healthcare system in the present pandemic, especially in developing countries [4]. In our case, the patient presented with AUFI, fulfilling the laboratory criteria for DF. He subsequently tested positive for COVID-19 disease through nasopharyngeal swab by RT-PCR, prompted by the new-onset breathlessness.

Since the COVID-19 pandemic is still ongoing in Asia, which is a DENV endemic zone, co-infection of DENV and SARS-CoV-2 virus could be tricky, thereby leading to delay in the diagnosis. Although both are ribonucleic acid (RNA) viruses, they have varied pathophysiology and target organ involvement but eventually lead in the similar direction of cytokine release, resulting in dysfunction in the integrity of the vascular endothelium leading to vasculopathy, coagulopathy, and capillary leak [5]. The phenomenon of antibody-dependent enhancement (ADE) has been described for both viruses, resulting in acceleration in the degree of infection and incidence of complications [6]. Various animal studies have also shown a possible role of the angiotensin-II converting enzyme (ACE) and angiotensin in the pathophysiology of viruses such as DENV, SARS-CoV-2, and H7N9 influenza [7].

The characteristics of both the viruses in terms of epidemiological and clinical presentation are shown in Table 2 [8]. A study by Yue et al. showed a huge overlap between clinical spectrums of both diseases, with fever and headache being the most common symptoms in both [2]. Our patient had presented with fever and bleeding manifestation with no respiratory and cardiovascular symptoms at presentation or no exposure to COVID-19, and hence the possibility for a tropical illness was significant. This calls for a high index of suspicion to test for other causes in the setting of one, especially in endemic areas during monsoon and post-monsoon seasons. Other than non-specific signs and symptoms, which present an unpredictable influence on the severity and clinical outcome of the patient, the co-infection presents with diagnostic challenges too. Serious precautions taken to prevent the spread of transmission of COVID-19 in a febrile patient can cause a potentially dangerous delay in the diagnosis of DF. Furthermore, the COVID-19 pandemic has led to global shortages in certain laboratory reagents. Since the molecular diagnosis of both COVID-19 and DENV requires the same extraction kits and enzymes, the depletion of stocks has escalated [9]. Misleading results due to cross-reaction between these two viruses have been reported in previous studies, demonstrating that antibodies to DENV envelope hypothetically binds to “receptor binding motif (RBM)” of the spike protein of SARS-CoV-2, with certain interactions diverting the binding of angiotensin-converting enzyme (ACE-2) receptor to RBM [10].

**Table 2:** Characteristic features of dengue fever and COVID-19 COVID-19: coronavirus disease 2019; HIV: human immunodeficiency virus; DENV: dengue virus; ICU: intensive care unit

	Characteristics	Dengue fever	COVID-19
1.	Incubation period	3-10 days, typically 5-7 days	~14 days, median of 4-5 days from symptom onset
2.	Signs and symptoms		
	Mild to moderate	Febrile phase: fever, headache with eye pain, myalgia, nausea, vomiting, rash, and leukopenia	Fever or chills, headache, myalgia, fatigue, sore throat, cough, shortness of breath, loss of taste or smell, rhinorrhoea, nausea, vomiting, and diarrhea
		With warning signs: abdominal pain or tenderness, persistent vomiting, clinical fluid accumulation, mucosal bleeding, lethargy, restlessness, and liver enlargement	
	Severe illness	DF with any of the following: plasma leakage leading to shock, fluid accumulation with respiratory distress, and severe bleeding with thrombocytopenia	Median time to dyspnea ranged from 5-8 days, the median time to acute respiratory distress syndrome (ARDS) ranged from 8-12 days, and the median time to ICU admission ranged from 10-12 days. Signs and symptoms of severe illness can include dyspnea, hypoxia, respiratory failure, shock, and multiorgan system dysfunction
		Severe organ impairment such as liver disease with elevated transaminases, or meningoencephalitis with impaired consciousness	
3.	Risk factors for severe illness	Age: infant; second DENV infection, chronic medical conditions including diabetes, asthma, or heart disease	Age >65, underlying conditions like cardiovascular disease, diabetes, chronic respiratory disease, hypertension, prior stroke, liver disease, obesity, chronic lung disease, chronic kidney disease undergoing dialysis, or immunocompromised status (i.e., poorly controlled HIV infection, undergoing cancer treatment, using corticosteroids, and smoking)
			People who live in a nursing home or long-term care facility

Apart from posing a diagnostic dilemma, the co-infection of DENV and COVID-19 also leads to some therapeutic quandaries. Hemodynamic instability in the case of DF requires fluid administration, but it should be done cautiously due to increased chances of developing pulmonary edema and worsening of oxygenation with COVID-19 infection. COVID-19 infection has been associated with increased chances of thrombosis, which requires the administration of low molecular-weight heparin (LMWH). This, however, puts the patient at risk of life-threatening bleeding manifestations due to thrombocytopenia as a result of DENV infection, especially with a platelet count of <1 lakh/mm^3^. The latest guidelines recommend close monitoring of D-dimer levels and platelet count if LMWH has to be given. Steroids have been shown to have a beneficial effect in COVID-19, with no negative repercussions for DF. Tocilizumab, antivirals, and other supportive management can be continued for COVID-19 as per the guidelines.

## Conclusions

Dual viral infection of COVID-19 and DENV is not uncommon; it is a demanding condition, which requires a high index of suspicion. The symptoms may be non-specific in most of the cases and an individual can be asymptomatic till late in the disease course. The treating physician should be familiar with these clinical scenarios. Preventive measures like social distancing and cough etiquette implementation should be coupled with vector control and the use of insect repellents inside the hospital too. A concerted effort should be undertaken to prevent, diagnose, and treat this co-infection using vigilant measures, especially in endemic areas during rainy and post-rainy seasons.
